# Prevalence of Infections and Antimicrobial Resistance of ESKAPE Group Bacteria Isolated from Patients Admitted to the Intensive Care Unit of a County Emergency Hospital in Romania

**DOI:** 10.3390/antibiotics13050400

**Published:** 2024-04-27

**Authors:** Alina-Simona Bereanu, Rareș Bereanu, Cosmin Mohor, Bogdan Ioan Vintilă, Ioana Roxana Codru, Ciprian Olteanu, Mihai Sava

**Affiliations:** 1 Faculty of Medicine, Lucian Blaga University of Sibiu, Lucian Blaga Street 2A, 550169 Sibiu, Romania; alina.bereanu@ulbsibiu.ro (A.-S.B.); bogdan.vintila@ulbsibiu.ro (B.I.V.); ioanaroxana.bera@ulbsibiu.ro (I.R.C.); mihai.sava@ulbsibiu.ro (M.S.); 2 County Clinical Emergency Hospital, Bld. Corneliu Coposu, nr. 2-4, 550245 Sibiu, Romania; ciprian.laborator@yahoo.com

**Keywords:** MDR bacteria, ESKAPE group, carbapenemase-producing *Klebsiella pneumoniae*, KPC-KP

## Abstract

The ESKAPE group (*Enterococcus faecium*, *Staphylococcus aureus*, *Klebsiella Pneumoniae*, *Acinetobacter baumannii*, *Pseudomonas aeruginosa*, *Enterobacter* spp.) is a group of bacteria very difficult to treat due to their high ability to acquire resistance to antibiotics and are the main cause of nosocomial infections worldwide, posing a threat to global public health. Nosocomial infections with MDR bacteria are found mainly in Intensive Care Units, due to the multitude of maneuvers and invasive medical devices used, the prolonged antibiotic treatments, the serious general condition of these critical patients, and the prolonged duration of hospitalization. Materials and Methods: During a period of one year, from January 2023 to December 2023, this cross-sectional study was conducted on patients diagnosed with sepsis admitted to the Intensive Care Unit of the Sibiu County Emergency Clinical Hospital. Samples taken were tracheal aspirate, catheter tip, pharyngeal exudate, wound secretion, urine culture, blood culture, and peritoneal fluid. Results: The most common bacteria isolated from patients admitted to our Intensive Care Unit was *Klebsiella pneumoniae*, followed by *Acinetobacter baumanii* and *Pseudomonas aeruginosa*. Gram-positive cocci (*Enterococcus faecium* and *Staphilococcus aureus*) were rarely isolated. Most of the bacteria isolated were MDR bacteria. Conclusions: The rise of antibiotic and antimicrobial resistance among strains in the nosocomial environment and especially in Intensive Care Units raises serious concerns about limited treatment options.

## 1. Introduction

The global spread of multidrug-resistant bacteria has led to increased in-hospital mortality and reduced treatment options, making it an increasingly worrisome problem. According to statistics, in 2019, 1.27 million deaths were directly caused by bacterial resistance to antibiotics, and 4.95 million deaths were associated with bacterial resistance [1,2]. The ESKAPE group (*Enterococcus faecium*, *Staphylococcus aureus*, *Klebsiella Pneumoniae*, *Acinetobacter baumannii*, *Pseudomonas aeruginosa*, *Enterobacter*) is a group of bacteria very difficult to treat, due to their high capacity to acquire resistance to antibiotics and which are the main causes of nosocomial infections worldwide, posing a threat to global public health [3]. The acronym is sometimes extended to ESKAPEE to also include *Escherichia coli* [4].

The worldwide spread of multidrug-resistant Gram-negative bacteria (MDR-GNB), especially *Klebsiella pneumoniae*, *Acinetobacter*, *Pseudomonas aeruginosa*, and *Enterobacter*, is a worrying threat. There is particular concern regarding the spread of multi-drug-resistant *Klebsiella pneumoniae* (MDR-KP). *Klebsiella pneumoniae* is a pathogenic, non-motile bacterium that possesses special abilities to acquire antimicrobial resistance due to the accumulation of mobile antimicrobial resistance genes gained through horizontal gene transfer. In the Intensive Care Units, it is frequently associated with ventilator-associated pneumonia and sepsis, possibly even leading to septic shock and death. There is no optimal antibiotic regimen; several combinations of antibiotics have been used (aminoglycosides, meropenem, colistin, tigecycline, fosfomycin, ceftazidime/avibactam) without satisfactory results. The main resistance mechanism of multi-resistant Gram-negative bacteria (MDR-GNB) is the production of enzymes (beta-lactamases, especially carbapenemases). Some bacteria (e.g., *Klebsiella pneumoniae*, *Escherichia coli*) produce extended-spectrum beta-lactamase (ESLB). Carbapenemases are the most potent Beta-lactamases and have versatile hydrolytic capacity against Beta-lactams (including carbapenems, cephalosporins, penicillin, and aztreonam). Three major classes of carbapenemases are involved: Ambler class A (*Klebsiella pneumoniae* carbapenemases) (KPC), B (Metallo-beta-lactamases) (MBLs), and D (oxacillinases) (OXA). These three groups of enzymes are plasmid-mediated, implying easy horizontal transfer. Mobile genetic elements involved in the coding of carbapenemases are associated with a high capacity to spread worldwide; the situation in many countries is not yet well documented [5,6,7,8]. The beta-lactamase (*bla*) mobile resistance gene encodes the enzyme that hydrolyzes carbapenems. This gene is distributed worldwide in various Gram-negative bacteria on several plasmids, in recombined and highly transposon-rich genomic regions, which is the cause of the global dissemination of MDR-GNB [1,8,9]. *Klebsiella pneumoniae* carbapenemase (KPC)-producing *Enterobacteriaceae* are endemic in Greece, Italy, Israel, the United States, Argentina, and Colombia. Those producing New Delhi Metallo-Beta-Lactamase-1 (MBL NMD-1) are endemic in India, Sri Lanka, and Pakistan, and those producing OXA-48-like oxacillinases are encountered in Malta, Turkey, the Middle East, and North Africa [9,10,11,12]. In 2016, the Annual Report of the European Antibiotic Surveillance Network reported an average carbapenem resistance percentage of 6.1%, with prevalent distribution in Greece, Romania, and Italy [12]. In 2022, the European Centre for Disease Prevention and Control and the National Public Health Organization in Greece published the results of their study, issuing a warning about the rapid spread of carbapenemase-producing, highly drug-resistant *Klebsiella pneumoniae* (sequence type 39) in hospitals in Greece [13]. A significant characteristic of *Klebsiella pneumoniae* virulence is the capacity to develop biofilms.

The resistance mechanisms of *Acinetobacter baumannii* are based on its genetic plasticity, which facilitates rapid genetic mutations and rearrangements. In addition, it easily forms biofilms. There are three main mechanisms that confer resistance to antibiotics: control of antibiotic transport (reduction of membrane permeability or increase in antibiotic efflux), modification of antibiotic targets, and enzymatic inactivation of antibiotics. *Acinetobacter baumannii* can form biofilms on medical devices, especially Intensive Care unit ventilators [14].

*Pseudomonas aeruginosa* exhibits three main mechanisms of antibiotic resistance: intrinsic resistance, decreased outer membrane permeability, and multidrug efflux systems. It also possesses the ability to easily colonize and form biofilms on medical devices and surfaces in the hospital environment [15].

The resistance mechanism of Gram-positive bacteria can occur by two major strategies: by the production of β-lactamases leading to the enzymatic degradation of the antibiotic or by decreasing the affinity and susceptibility of the target site, namely the penicillin-binding protein (PBP), either by acquiring exogenous DNA or by modifications of native PBP genes. Among Gram-positive bacteria of global concern are Methicillin-resistant *Staphylococcus aureus* (MRSA) and Vancomycin-resistant *Enterococcus faecium* (VRE) [16,17].

Antimicrobial resistance (AMR) is a public health problem. The World Health Organization (WHO) launched the Global Antimicrobial Resistance and Use Surveillance System (GLASS). Despite limitations in some countries related to staff shortages, data management issues, budget issues, and reduced laboratory capacity, an increasing number of countries are working to report data more fully and efficiently, expand their surveillance capacity, and combat antimicrobial resistance (AMR) [18].

In recent years, the guidelines have focused on the implementation of measures to control and prevent nosocomial infections in hospitals, but the effectiveness of these measures remains unknown. However, there is still a lack of consensus regarding the effectiveness of individual control measures regarding the spread of MDR bacteria [19,20,21,22,23,24]. These germs are capable of persisting in human reservoirs, in the intrahospital environment, on medical devices and equipment, and generating biofilms resistant to antimicrobial substances and general disinfection measures. Increased resistance is mainly due to the limited diffusion of antibiotics and antimicrobial substances through the biofilm matrix, but also to the phenotypic and genotypic characteristics of biofilm microorganisms that differ from those of planktonic microorganisms [25,26,27,28].

Bacteria are unicellular organisms. Bacterial biofilms are aggregates of bacteria of the same or different species embedded in the extracellular polymeric substance (EPS) produced by them, adherent to each other and to biotic or abiotic surfaces. Pathogenic agents colonize tissues, medical surfaces, and medical devices, reaching, under certain conditions, to form mature biofilms that protect the inside bacteria from hostile factors such as the host’s immune response, environmental factors (UV radiation, extreme pH, extreme temperature, high salinity, high pressure), and antimicrobial agents (antibiotics, disinfectants). The steps of bacterial biofilm formation are as follows: adhesion of the bacterium to the surface, irreversible attachment (secretion of EPS and inhibition of motility factor), maturation of the biofilm, and dispersal of the bacteria from the biofilm (the bacteria returning to their original form). As a final result, the biofilm expands and establishes itself in new places, contributing to the dissemination of bacteria, disease progression, and transmission of infection [29,30,31,32,33,34,35,36]. A mature biofilm can contain over 100 trillion bacterial cells per milliliter. These cells communicate with each other through autoinductive signals; this communication is essential for biofilm development because the inside bacteria are organized into different communities, and each community has a specific task [30,37,38].

A total of 60–80% of microbial infections are related to the formation of bacterial biofilms [38,39,40]. The most common bacteria involved in infections associated with biofilms on medical devices are *Staphylococcal* species (*S. epidermidis*, *S. aureus*) and multidrug-resistant Gram-negative bacteria (especially *K. pneumoniae* and *P. aeruginosa*, *A. baumannii*, *E. coli*). *Staphylococcus aureus* biofilms are involved in the following infections: endocarditis, chronic otitis media, chronic rhinosinusitis, chronic osteomyelitis, and post-orthopedic implant infections [30]. *Escherichia coli* biofilms are involved in acute and recurrent urinary tract infections and biliary tract infections [30,41]. *Pseudomonas aeruginosa* biofilms are involved in cystic fibrosis lung infection, chronic wound infection, chronic rhinosinusitis, chronic otitis media, burn wound infection, catheter-associated urinary infection, and contact lens-related keratitis [30,42]. The most common microorganisms isolated from patients with ventilator-associated pneumonia (VAP) are Gram-negative aerobic bacteria (*Klebsiella pneumoniae*, *Acinetobacter species*, *Pseudomonas aeruginosa*, *Enterobacter* spp., *Serratia mercescens*, and *Stenotrophomonas maltophilia*), in over 60% of cases, and Gram-positive cocci (especially methicillin-resistant *Staphylococcus aureus*—MRSA) [33,43,44,45,46].

Regarding the microorganisms associated with biofilms isolated from medical devices, associations were described in which bacteria from the ESKAPE group are frequently found: *Enterococci*, *Staphylococcus aureus* at the level of cardiac valve prostheses; *Staphylococcus aureus*, *Enterococcus faecalis*, *Pseudomonas aeruginosa*, *Klebsiella pneumoniae* at the level of central venous catheters; *Escherichia coli*, *Klebsiella pneumoniae*, *Enterococcus faecalis*, at the level of urinary catheters; *Staphylococcus aureus*, *Enterococcus* species at the level of intrauterine devices; *Enterococci*, *Staphylococcus aureus*, and *Escherichia coli* in hip prostheses [30].

The risk factors for colonization and infections with MDR germs are voyages to endemic areas, inadequate antibiotic therapy, prolonged hospitalization in Intensive Care Units, dependence on invasive medical devices, and the presence of comorbidities (e.g., neoplasia, diabetes, chronic obstructive pulmonary disease) [11,47,48,49,50]. Nosocomial infections with MDR bacteria are found mainly in Intensive Care Units due to the multitude of maneuvers and invasive medical devices used, the prolonged antibiotic treatments, the serious general condition of these critical patients, and the prolonged duration of hospitalization. Mortality in these patients remains high despite the discovery and use of new antibiotics and antimicrobials. For example, the mortality rate of patients with bacteremia or respiratory infections caused by carbapenemase-producing *Klebsiella pneumoniae* varies between 30 and 70% [12,13,51,52,53].

In our intensive care unit, we also face this major problem, with the presence of MDR bacterial infections from the ESKAPE group, with the KPC-KP being one of the most frequently isolated bacteria from samples collected from critical patients in our ward.

## 2. Results

**Figure 1:**
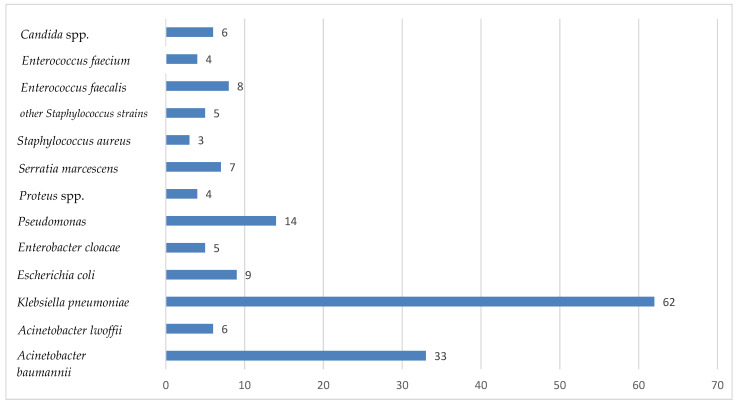
Microorganisms isolated from samples collected from patients with healthcare-associated infections in the Intensive Care Unit within the Sibiu County Emergency Clinical Hospital between 1 January 2023 and 31 December 2023—(*x* axis—number of positive samples).

Over the period of one year (from 1 January 2023 to 31 December 2023), the prevalence of nosocomial infections with bacteria from the ESKAPEE group in the Intensive care unit of the Sibiu County Emergency Clinical Hospital and the susceptibility of these bacteria to antibiotics were monitored. In total, 160 bacterial strains and six fungal strains (*Candida*) were isolated. Among the types of bacteria isolated, 75.6% were bacteria belonging to the ESKAPE group; the other bacteria isolated were *Enterococcus faecalis*, other *Staphylococcus* strains, *Serratia marcescens*, *Proteus* spp., *Escherichia coli* and *Acinetobacter lowffii*. The most common microorganism isolated from patients admitted to our Intensive Care Unit was *Klebsiella pneumoniae* (38.7%), followed by *Acinetobacter baumannii* (20.6%) and *Pseudomonas aeruginosa* (8.7%), *Escherichia coli* (5.6%), and *Enterobacter cloacae* (3.1%). Gram-positive cocci were rarely isolated: *Enterococcus faecium* (2.5%) and *Staphylococcus aureus* (1.8%) (Figure 1).

**Figure 2:**
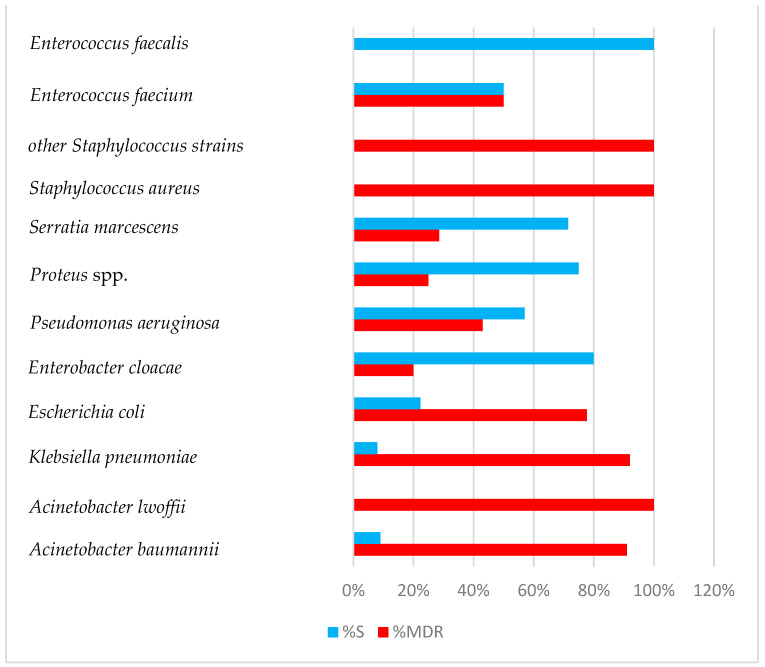
The percentage of MDR strains from the total number of bacterial strains isolated from samples collected from patients with healthcare-associated infections between 1 January 2023 and 31 December 2023 (Intensive care unit of Sibiu County Clinical Emergency Hospital, Romania) MDR (multidrug-resistant), S (sensitivity).

Figure 2 shows the percentage of MDR bacteria. As can be seen, the ESKAPE group has a worrying percentage of MDR bacteria.

**Figure 3:**
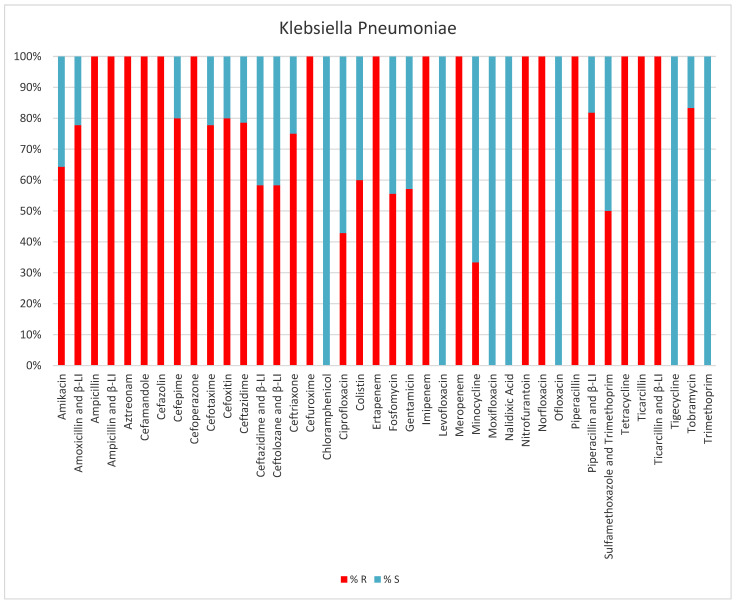
Results of antibiotic susceptibility testing of *K. pneumoniae* isolated from critically ill patients between January and December 2023 (Intensive Care Unit of the Sibiu County Clinical Emergency Hospital, Romania) S (sensitivity), R (resistance), β-LI (beta-lactamase inhibitor).

Between January and December 2023, we had 62 *Klebsiella pneumoniae* infections in critical patients; most of the samples (26 samples) (41.9%) were from tracheal aspirate (intubated and mechanically ventilated patients). Other samples positive for *K. pneumoniae* were the central venous catheter tip (8%), urine (16.1%), blood (17.7%), superinfected surgical wounds, abscesses, and drain tubes (12.9%). The majority of *K. pneumoniae* strains (91.9%) were resistant to antibiotics (ESLB, MDR, XDR, and even PDR strains). Five isolated strains (8%) were PDR *Klebsiella pneumoniae.* Only five strains (8%) of those isolated did not present a resistance pattern (Table 1, Figure 3).

**Figure 4:**
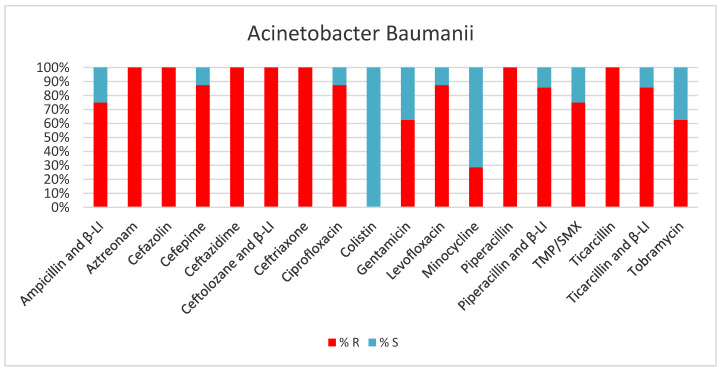
Results of antibiotic susceptibility testing of *Acinetobacter Baumannii* isolated from critically ill patients between January and December 2023 (Intensive Care Unit of the Sibiu County Clinical Emergency Hospital, Romania). β-LI (beta-lactamase inhibitor), TMP/SMX (Trimethoprim and Sulfamethoxazole).

In 2023, *Acinetobacter baumannii* was the second most prevalent microorganism isolated in our department, accounting for 20.6% of all positive samples. Notably, tracheal secretions from intubated and mechanically ventilated patients were the most common source of this microorganism, with a prevalence of 54.5%. Moreover, a significant proportion of these strains exhibited multidrug-resistant (MDR) and extensively drug-resistant (XDR) phenotypes, posing significant challenges to effective treatment (Table 2, Figure 4).

**Figure 5:**
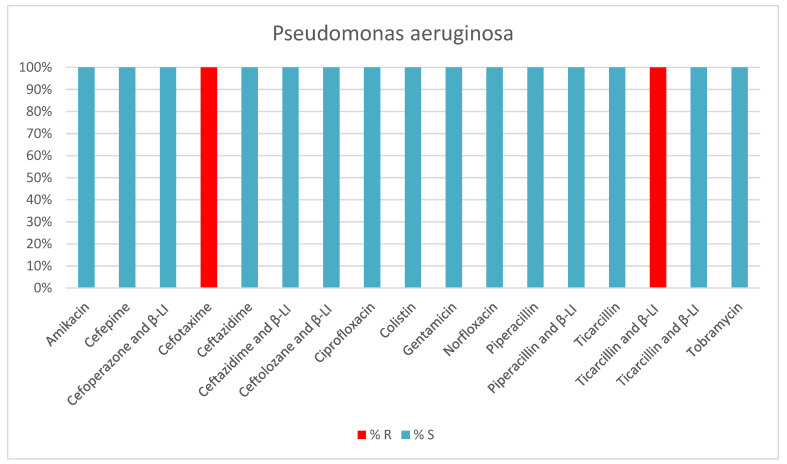
Results of antibiotic susceptibility testing of P. aeruginosa isolated from critically ill patients between January and December 2023 (Intensive Care Unit of the Sibiu County Clinical Emergency Hospital, Romania) S (sensitivity), R (resistance), β-LI (beta-lactamase inhibitor).

*Pseudomonas aeruginosa* was present in 8.7% of bacteria-positive samples collected from patients; most *Pseudomonas aeruginosa*-positive samples (64.2%) were also from tracheal aspirates of intubated and mechanically ventilated patients. A total of 42.8% of *Pseudomonas aeruginosa* strains were MDR and XDR (Table 3, Figure 5).

**Figure 6:**
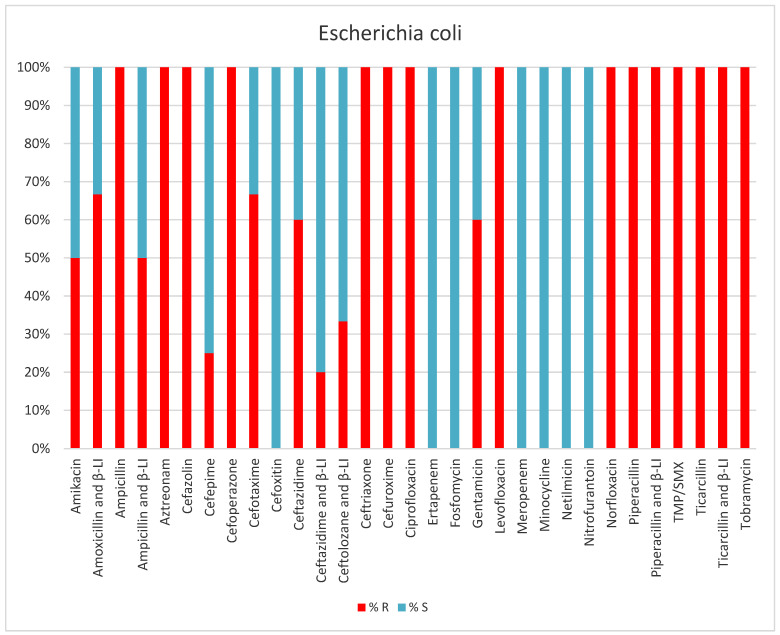
Results of antibiotic susceptibility testing of *E. coli* isolated from critically ill patients between January and December 2023 (Intensive Care Unit of the Sibiu County Clinical Emergency Hospital, Romania) S (sensitivity), R (resistance), β-LI (beta-lactamase inhibitor), TMP/SMX (Trimethoprim and Sulfamethoxazole).

*Escherichia coli* was present in 5.6% of the collected samples, mostly from urine (55.5%) and ESLB-positive (Table 4, Figure 6).

**Figure 7:**
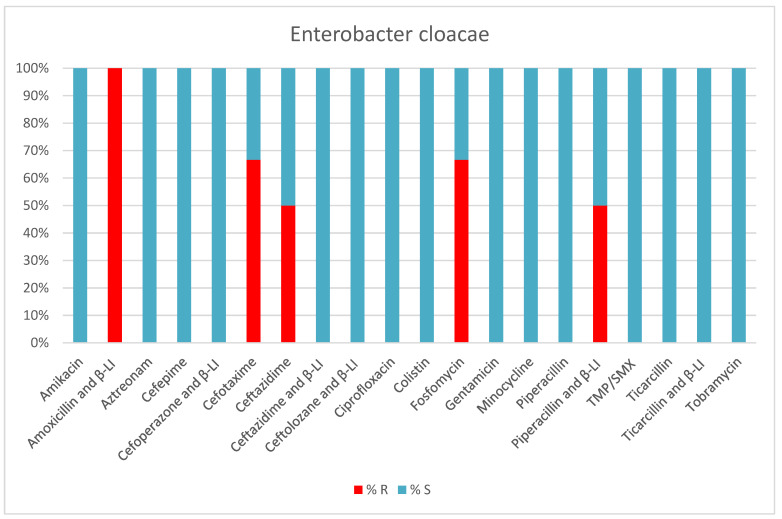
Results of antibiotic susceptibility testing of *Enterobacter cloacae* isolated from critically ill patients between January and December 2023 (Intensive Care Unit of the Sibiu County Clinical Emergency Hospital, Romania) S (sensitivity), R (resistance), β-LI (beta-lactamase inhibitor), TMP/SMX (Trimethoprim and Sulfamethoxazole).

*Enterobacter cloacae* was present in 3.1% of bacteria-positive samples collected from patients. It was most frequently found in tracheal secretions of intubated and mechanically ventilated patients. A total of 20% of *Enterobacter cloacae* strains were ESLB-producing (Table 5, Figure 7). 

**Figure 8:**
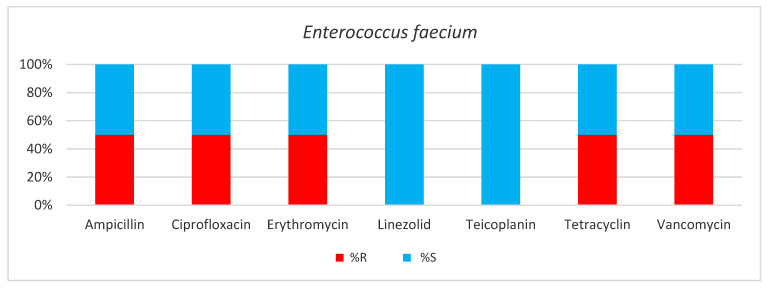
Results of antibiotic susceptibility testing of *Enterococcus faecium* isolated from critically ill patients between January and December 2023 (Intensive Care Unit of the Sibiu County Clinical Emergency Hospital, Romania) S (sensitivity), R (resistance).

*Enterococcus faecium* was present in 2.5% of positive bacteria samples collected from our Intensive Care patients. It was isolated from superinfected surgical wounds and abscesses (in 75% of cases) and from urine (25%). A total of 50% of the strains belonged to the Vancomycin-resistant *Enterococcus* group (VRE) (Table 6, Figure 8).

**Figure 9:**
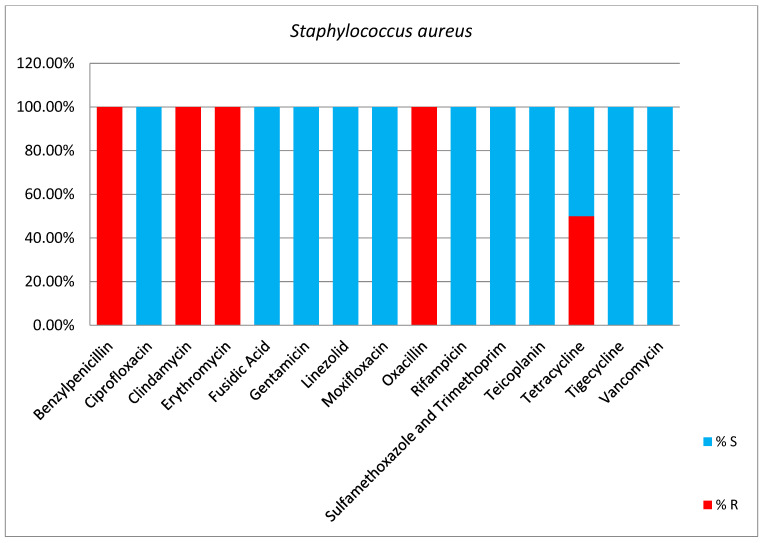
Results of antibiotic susceptibility *Staphylococcus aureus* isolated from critically ill patients between January and December 2023 (Intensive Care Unit of the Sibiu County Clinical Emergency Hospital, Romania) S (sensitivity), R (resistance).

*Staphylococcus aureus* was less common in our patients (1.8% of isolated bacteria). All strains were methicillin-resistant *Staphylococcus aureus* (MRSA), isolated from tracheal aspirate (33.3%), blood (33.3%), and surgical wounds (33.3%) (Table 7, Figure 9).

As a synthesis of the data, the ESKAPE group bacteria were mainly isolated from the tracheal aspirate secretions of intubated and ventilated patients. The leading cause of sepsis in patients intubated and mechanically ventilated for more than 48 h is ventilator-associated pneumonia (VAP). The most common microorganism identified in tracheal aspirates was *Klebsiella pneumoniae* (found in 43.33% of all positive tracheal aspirates), followed by *Acinetobacter baumannii* (30%) and *Pseudomonas aeruginosa* (15%). Many of the isolated strains demonstrated resistance to antibiotics, with some being multidrug-resistant (MDR), extensively drug-resistant (XDR), and some even pan-drug-resistant (PDR). Gram-positive cocci were rarely isolated from tracheal aspirates. *Staphylococcus aureus* MRSA was present in 1.66% of all tracheal aspirates positive for bacteria. *Escherichia coli* predominated in urine (55.5%) and was also isolated from tracheal secretions and superinfected surgical wounds. *Enterococcus faecium* was isolated from superinfected surgical wounds and urine, and Methicillin-resistant *Staphylococcus aureus* (MRSA) was equally present in tracheal aspirate, surgical wounds, and blood.

There was no correlation between the antibiotic resistance profile and the anatomical site from which the bacteria was isolated. Thus, *Klebsiella pneumoniae* was mainly isolated from tracheal secretions, and most isolates showed antibiotic resistance (MDR, XDR, or PDR). Only 19.23% of *Klebsiella pneumoniae* strains isolated from tracheal aspirates were without a resistance pattern. *Klebsiella pneumoniae* has also been isolated from pharyngeal exudate, urine, blood, central venous catheter tips, surgical wounds, abscesses, and abdominal drains, all of which showed antibiotic resistance (MDR, XDR, PDR). The second most common microorganism isolated from tracheal aspirates was *Acinetobacter baumannii*, with most of these strains showing resistance to antibiotics (MDR and XDR). Only 16.66% of *Acinetobacter baumannii* strains isolated from tracheal aspirate did not present a resistance pattern. *Acinetobacter baumannii* was also present in pharyngeal exudate, peritoneal fluid, superinfected surgical wounds, blood, and urine, all MDR or XDR strains.

There were differences in the antibiotic resistance profile between different species of bacteria. From the class of Gram-negative bacteria, *Klebsiella pneumoniae* and *Acinetobacter pneumoniae* showed the highest antibiotic resistance. A total of 91.9% of *K. pneumoniae* strains were ESLB, MDR, XDR, and PDR. A total of 91% of *Acinetobacter baumanni* strains were MDR and XDR strains. Resistance to all antibiotics tested (PDR) was present only in some strains of *Klebsiella pneumoniae* (8% of all *K. pneumoniae* strains) isolated from tracheal aspirates, central venous catheter tips, and superinfected surgical wounds. In the case of *Pseudomonas aeruginosa*, 42.8% of the strains were MDR strains. Only 20% of *Enterobacter cloacae* strains were ESLB-positive. In the group of Gram-positive cocci, all strains of *Staphylococcus aureus* isolated from different sites were MRSA. Regarding *Enterococcus faecium*, 50% of strains showed resistance to Vancomycin.

## 3. Discussion

In our Intensive Care Unit, we are dealing with nosocomial infections with bacteria from the ESKAPE group (75.6%). Several studies describe the ability of ESKAPE group bacteria to form biofilms on biotic and abiotic surfaces, and these biofilms lead to increased resistance to exogenous antimicrobial factors and antibiotics. Bacterial biofilms constitute important reservoirs of multi-drug-resistant pathogenic bacteria [54,55,56,57,58,59,60,61,62,63,64,65,66,67,68,69]. These are of two types: hydrated biofilms (for example, in drains, catheters, and parts of some medical devices) and dry surface biofilms (DSB) (on surfaces and some medical devices). MDR Gram-negative bacteria, especially *K. pneumoniae*, *Acinetobacter baumannii*, *P. aeruginosa*, and *E. coli* are most commonly present in urinary catheter and central venous catheter (CVC) biofilms [28,30,32]. In their study, Folliero and colleagues (2021) isolated the main bacteria contaminating medical devices and studied their ability to form biofilms and the prevalence of MDR strains in biofilms. Thus, they found that *K. pneumoniae* was frequently present in CVCs, urinary catheters, nephrostomy tubes, and abdominal drains. A total of 72.7% of *K. pneumoniae* strains were biofilm producers. They isolated more than one microorganism from some medical devices. Regarding antibiotic susceptibility, 59.2% of the isolated strains were MDR strains [28]. In our ward as well, *Klebsiella pneumoniae* was the most frequently isolated microorganism from patient secretions or from invasive medical devices fitted to critical patients (e.g., CVCs, abdominal drains) (39% of isolated strains belonged to *K. pneumoniae*). In our study, *K. pneumoniae* was isolated most frequently from tracheal secretions (tracheal aspirate) collected from mechanically ventilated critically ill patients. Regarding antibiotic susceptibility, 91.9% of the *K. pneumoniae* strains isolated from our ward showed resistance to antibiotics (ESLB, MDR, XDR, and even PDR strains). The risk factors that caused multi-drug-resistant *Klebsiella pneumoniae* to become prevalent in our department are the multitude of maneuvers and medical devices used, given that we are an Intensive Care Unit, as well as the frequent and long-term use of antibiotics and antimicrobials, the large number of days of hospitalization, patients with serious general conditions and the frequent presence of comorbidities in these patients. In addition, we take into consideration the epidemiological factor, the spread of *Klebsiella pneumoniae* carbapenemase (KPC)-producing *Enterobacteriaceae* in Europe and the prevalence of carbapenem resistance in Romania, along with Greece and Italy [9,10,11,12,13].

Many nosocomial infections are infections associated with biofilms at the level of catheters (hydrated biofilms): urinary tract infections associated with urinary catheters, bloodstream infections associated with central venous catheters, and respiratory infections associated with intubation tubes or tracheal cannulas. One of the most common and severe infections in critically intubated and ventilated Intensive Care Unit patients is ventilator-associated pneumonia (VAP). This occurs in patients intubated and ventilated for at least 48 h. The etiology is usually bacterial, with Gram-negative bacteria (*Klebsiella pneumoniae*, *Acinetobacter species*, *Pseudomonas aeruginosa*, *Enterobacter* spp., *Stenotrophomonas maltophilia*, *Serratia marcescens*) in 60% of cases and Gram-positive cocci (especially methicillin-resistant *Staphylococcus aureus*—MRSA) [33,43,44,45,46]. In our ward as well, the most frequent bacteria isolated from patients belonged to the class of Gram-negative bacteria, accounting for 76.7% of the total isolated bacteria. Among them, the most frequently encountered was *Klebsiella pneumoniae* (38.7% of the total bacteria isolated), followed by *Acinetobacter baumannii* (20.6%), *Pseudomonas aeruginosa* (8.7%), *Escherichia coli* (5.6%), and *Enterobacter cloacae* (3.1%). These microorganisms were isolated from our critically ill patients dependent on invasive medical devices (endotracheal tubes, urinary tubes, drain tubes), where these bacteria can easily form biofilms. Gram-positive cocci were less involved in nosocomial infections in our intensive care unit (*Enterococcus faecium* 2.5% and *Staphylococcus aureus* 1.8%).

The materials from which catheters and invasive medical devices are made can influence the adhesion of bacteria and the formation of bacterial biofilms on them. Thorarinsdottir and colleagues (2020) described in their prospective study the importance of the material from which endotracheal tubes are made. Thus, they demonstrated that the noble-metal-coated polyvinil chloride (PVC) endotracheal tubes were associated with a lower rate of bacterial biofilm formation compared to plain PVC ones [70]. One of the essential factors in the adhesion of microorganisms to surfaces is hydrophobicity. Microbes tend to adhere to surfaces because of the nature of their cell walls. Hydrophobic cells have a stronger affinity for hydrophobic surfaces, whereas hydrophilic cells adhere more strongly to hydrophilic surfaces. Metal is hydrophilic and PVC is hydrophobic, which explains the stronger adherence of hydrophobic bacteria to PVC endotracheal probes compared to those composed of noble-metal-coated PVC. The high degree of adherence and biofilm formation of *K. pneumoniae* on abiotic surfaces may be attributed to the hydrophobic nature of the bacterial surface [71,72,73,74]. The probes used in our department are made of PVC. The most common microorganism isolated in our department was *K. pneumoniae* and it was most frequently present in tracheal secretions (42%) in mechanically ventilated patients. 

MDR bacteria easily form biofilms on invasive medical devices (catheters, drain tubes, endotracheal tubes), but also on the hands of medical personnel and on surfaces in the hospital environment [28,32,33,70,71,72,73,74,75,76,77]. Adhesion to the surface is a crucial step in bacterial growth. Bacterial resistance to antimicrobials (including antibiotics) occurs in the hospital environment because pathogens remaining after insufficient antibacterial treatment proliferate. Repeating this process leads to more bacteria becoming resistant to more antimicrobials, allowing proliferation, which, combined with their ability to form biofilms, makes them even more resistant. Bacteria develop defense mechanisms against antimicrobials either through genetic mutation or through the acquisition of genetic material through horizontal gene transfer within biofilms. On the other hand, the widespread use of antimicrobials leads to an increase in bacterial resistance [78,79,80].

Persistence in the hospital environment is a public health problem. Dry surface biofilms (DSB) present in the hospital environment (door handles, light switches, trolley handles, ceilings, curtains, keyboards, ventilator inlets, mattresses, and bed rails) are difficult to remove due to resistance to common disinfectants and antimicrobials and contribute to colonization, infection, and the spread of infections in the hospital [81,82,83,84,85]. In 2015, Hu and colleagues reported in their study that MDR bacteria are able to survive on contaminated surfaces despite disinfection with chlorine solution. They demonstrated that biofilms are polymicrobial in 93% of cases [83]. Polymicrobial biofilms are more resistant to disinfection than monomicrobial ones [84,86]. Dry surface biofilms, especially high-touch surfaces, constitute an important reservoir of pathogens [83]. In 2023, Centeleghe and colleagues published the first study confirming the ability of *Klebsiella pneumoniae* to survive for a long time on dry surfaces as a dry surface biofilm (DSB). The viability of this bacterium remains high at 4 weeks on a dry surface, even though culturability is low [87].

## 4. Materials and Methods

This study was performed with the approval of the Ethics Commission of the Sibiu County Emergency Clinical Hospital, Romania. During a period of one year, from January 2023 to December 2023, this cross-sectional study was conducted on patients admitted to the intensive care unit within the Sibiu County Emergency Clinical Hospital.

### 4.1. Sampling and Bacterial Isolation

The samples taken were tracheal aspirate (from intubated and ventilated patients), central venous catheter tip (extracted from the patient), pharyngeal exudate, superinfected surgical wound secretion, postoperative abscesses, fluid from drain tubes, urine culture (from patients with urine probes), blood culture, and peritoneal fluid from critically ill patients diagnosed with sepsis or septic shock admitted to our Intensive Care Unit.

Bacterial species were identified using classic biochemical tests (triple sugar iron TSI, motility indole urea MIU, Simmons citrate), Gram staining, and methylene blue, as well as automatic identification on the Vitek 2 Compact analyzer. In some situations, syndromic molecular biology tests from the upper respiratory tract were also used.

### 4.2. Antimicrobial Susceptibility Testing

The antibiogram, or testing the sensitivity (susceptibility) of a bacterial strain to antimicrobial agents, is one of the most frequent examinations requested in the bacteriology laboratory. Technically, there are 2 possibilities for testing the antibacterial activity;

- The Kirby-Bauer disk diffusion susceptibility test;
- The dilution method—allows the precise establishment of the minimum inhibitory concentration (MIC).

Kirby-Bauer disk diffusion method: the principle of the method consists of seeding the strain to be investigated on the surface of a solid Mueller Hinton medium and applying microtablets with antibiotic to its surface, according to the CLSI standard. They will diffuse into the agar from close to close, reaching lower and lower concentrations as they move away from the disk; the bacteria will grow in a “network” on the surface of the agar up to the area where the antibiotics will reach a concentration equal to the MIC. Their diameter is analyzed and compared with the diameters from the international standard (CLSI); depending on this, they fall into a certain category of sensitivity/resistance.

Vitek 2 Compact automatic antibiogram—allows identification and antibiotic sensitivity testing of bacteria and fungi isolated from clinical samples. The tests are carried out by means of cards available to the automatic system. For antibiotic sensitivity testing, the reading method of the device is turbidimetric. The device has software called Advanced Expert System Version 9.03 (AES), which is integrated into the system and validates the results of identification and antibiotic testing of bacteria by comparison with the extensive database of the advanced expert system (AES).

Antibiogram by broth microdilution method—the standardized method for testing the antibiotic sensitivity of germs. In our laboratory, it is used only for cases of MDR, XDR, and PDR infections. In the above-mentioned period, it was used only for colistin testing.

Strains that are resistant to at least one antibiotic from at least three antimicrobial classes were defined as MDR. Those resistant to at least one antibiotic from all antimicrobial categories, with the exception of one or two, were considered XDR, and strains resistant to all antimicrobial classes were defined as PDR.

## 5. Conclusions

MDR bacteria are frequently encountered in Intensive Care Units. Our Intensive Care Unit is facing this problem, too. MDR bacteria from the ESKAPE group were isolated from our patients with various infections in a high percentage (75.6% of the total bacteria isolated). By far, the most common microorganism in our ward was MDR *Klebsiella pneumoniae* (38.7%), followed by *Acinetobacter baumannii* (20.6%) and *Pseudomonas aeruginosa* (8.7%). Gram-positive cocci were less common: *Enterococcus faecium* was encountered in 2.5% of the samples (of which 50% were VRE) and *Staphylococcus aureus* in 1.8% of the total strains isolated from our patients (all strains were MRSA).

*Enterobacteriaceae* from the ESKAPE group were present mainly in secretions from tracheal aspirates, from intubated and mechanically ventilated patients, with ventilator-associated pneumonia being one of the most common nosocomial infections in Intensive Care Units, including our unit. *Escherichia coli* was mainly isolated from urine.

Gram-positive cocci were less involved in nosocomial infections in our Intensive Care Unit (*Enterococcus faecium* 2.5% and *Staphylococcus aureus* 1.8%). *Enterococcus faecium* was isolated from superinfected surgical wounds and urine, and Methicillin-resistant *Staphylococcus aureus* (MRSA) was equally present in tracheal aspirate, surgical wounds, and blood.

Most of the bacteria in the ESKAPE group were multidrug-resistant (less *Enterobacter cloacae*). There was no correlation between the antibiotic resistance profile and the anatomical site from which the bacteria was isolated. There were differences in the antibiotic resistance profile between different species of bacteria. *Klebsiella pneumoniae* and *Acinetobacter pneumoniae* showed the highest antibiotic resistance. A total of 91.9% of *K. pneumoniae* strains were ESLB, MDR, XDR, and PDR, while 91% of *Acinetobacter baumannii* strains were MDR and XDR strains. Resistance to all antibiotics tested (PDR) was present only in some strains of *Klebsiella pneumoniae* (8% of all *K. pneumoniae* strains) isolated from tracheal aspirates, central venous catheter tips, and superinfected surgical wounds. Increasing antibiotic and antimicrobial resistance among strains in the hospital environment raises serious concerns about limited treatment options.

## Figures and Tables

**Table 1:** *K. pneumoniae* strains isolated from critically ill patients between 1 January 2023 and 31 December 2023 (Intensive Care Unit of the Sibiu County Clinical Emergency Hospital, Romania) MDR (multidrug-resistant), XDR (extensively drug-resistant), PDR (pandrug-resistant).

*Klebsiella pneumoniae*	62
tracheal aspirate	26
without resistance pattern	5
Positive-MDR	4
Positive-ESBL	3
Positive-PDR	2
Positive-XDR	12
Catheter-tip	5
Positive-PDR	2
Positive-XDR	2
Positive -MDR	1
Pharyngeal exudate	2
Positive-XDR	2
Surgical wound, abscess, ulcer, etc.	8
Positive-PDR	1
Positive-XDR	6
Positive MDR	1
Blood	11
Positive-ESLB	2
Positive-MDR	1
Positive-XDR	8
Urine	10
Positive-MDR	4
Positive-XDR	6

**Table 2:** *Acinetobacter baumannii* strains isolated from critically ill patients between 1 January 2023 and 31 December 2023 (Intensive Care Unit of the Sibiu County Clinical Emergency Hospital, Romania) MDR (multidrug-resistant), XDR (extensively drug-resistant), PDR (pandrug-resistant).

*Acinetobacter baumannii*	33
tracheal aspirate	18
without resistance pattern	3
Positive-MDR	4
Positive-XDR	11
Pharyngeal exudate	5
Positive-MDR	1
Positive-XDR	4
Peritoneal fluid	2
Positive-MDR	1
Positive-XDR	1
Surgical wound, abscess, ulcer, etc.	3
Positive-MDR	1
Positive-XDR	2
Blood	2
Positive-XDR	2
Urine	3
Positive-XDR	3

**Table 3:** *P. aeruginosa* strains isolated from critically ill patients between 1 January 2023 and 31 December 2023 (Intensive Care Unit of the Sibiu County Clinical Emergency Hospital, Romania) MDR (multidrug-resistant).

*Pseudomonas aeruginosa*	14
Tracheal aspirate	9
without resistance pattern	4
Positive-MDR	5
Pharyngeal exudate	1
without resistance pattern	1
Surgical wounds, abscesses, ulcers, etc.	3
without resistance pattern	2
Positive-MDR	1
Urine	1
without resistance pattern	1

**Table 4:** *Escherichia coli* strains isolated from critically ill patients between 1 January 2023 and 31 December 2023 (Intensive Care Unit of the Sibiu County Clinical Emergency Hospital, Romania) ESLB (extended-spectrum beta-lactamase), XDR (extensively drug-resistant).

*Escherichia coli*	9
Tracheal aspirate	3
without resistance pattern	1
Positive-ESBL	1
Positive-XDR	1
Surgical wounds, abscesses, ulcers, etc.	1
without resistance pattern	1
Urine	5
Positive-ESBL	5

**Table 5:** *Enterobacter cloacae* strains isolated from critically ill patients between 1 January 2023 and 31 December 2023 (Intensive Care Unit of the Sibiu County Clinical Emergency Hospital, Romania) ESLB (extended-spectrum beta-lactamase).

*Enterobacter cloacae*	5
Tracheal aspirate	3
without resistance pattern	2
Positive-ESBL	1
Surgical wounds, abscesses, ulcers, etc.	1
without resistance pattern	1
Blood	1
without resistance pattern	1

**Table 6:** *Enterococcus faecium* strains isolated from critically ill patients between 1 January 2023 and 31 December 2023 (Intensive Care Unit of the Sibiu County Clinical Emergency Hospital, Romania) VRE (Vancomycin-resistant *Enterococcus faecium*).

*Enterococcus faecium*	4
Surgical wounds, abscesses, ulcers, etc.	3
negative VRE	1
positive-VRE	2
Urine	1
negative VRE	1

**Table 7:** *Staphylococcus aureus* strains isolated from critically ill patients between 1 January 2023 and 31 December 2023 (Intensive Care Unit of the Sibiu County Clinical Emergency Hospital, Romania) MRSA (Methicillin-resistant *Staphylococcus aureus*).

*Staphylococcus aureus*	3
Tracheal aspirate Positive-MRSA	1
Blood Positive-MRSA	1
Surgical wounds, abscesses, ulcers, etc. Positive-MRSA	1

## Data Availability

The manuscript contains all the data. Further information can be provided at the request of the Editor in chief.

## Abbreviations

AES	Advanced Expert System
β-LI	beta-lactamase inhibitor
AMR	Antimicrobial resistance
CLSI	Institute for Clinical and Laboratory Standards
CVC	Central venous catheter
CRKP	Carbapenem-resistant *Klebsiella pneumoniae*
DSB	Dry surface biofilms
EPS	Extracellular polymeric substances
ESLB	Extended spectrum beta-lactamase
GLASS	Global Antimicrobial Resistance and Use Surveillance System
ICU	Intensive care unit
KPC-KP	Carbapenemase-producing *Klebsiella pneumoniae*
MDR	Multidrug-resistant
MIC	Minimum inhibitory concentration
MIU	Motility indole urea
MRSA	Methicillin-resistant *Staphylococcus aureus*
PDR	Pandrug-resistant
PVC	Polyvinil chloride
R	Resistance
S	Sensitivity
TMP/SMX	Trimethoprim and Sulfamethoxazole
TSI	Triple sugar iron
VAP	Ventilator-associated pneumonia
VISA	Vancomycin-intermediate *Staphylococcus aureus*
VRE	Vancomycin-resistant *Enterococcus faecium*
VRSA	Vancomycin-resistant *Staphylococcus aureus*
WHO	World Health Organization
XDR	Extensively drug-resistant
